# XBP-1S TRANSCRIPTIONAL TARGETS IN THE ER UNFOLDED PROTEIN RESPONSE RESCUE AGING-RELATED TAUOPATHY PROTEOSTASIS

**DOI:** 10.1093/geroni/igae098.2829

**Published:** 2024-12-31

**Authors:** Brian Kraemer

**Affiliations:** VA Puget Sound Healthcare System, Seattle, Washington, United States

## Abstract

Proteostasis mechanisms fail with aging, promoting toxic protein accumulation. Neurons are particularly vulnerable to proteostatic disruption leading to aging related neurodegeneration. Abnormal activation of the endoplasmic reticulum unfolded protein response (UPRER) is implicated in tauopathies, a group of neurodegenerative diseases characterized by pathological accumulation of the microtubule-associated protein tau. Previous work showed neuronal overexpression of UPRER transcription factor XBP-1s suppresses tauopathy in C. elegans. Whole-genome RNA sequencing of C. elegans overexpressing XBP-1s showed upregulation of the following genes with human homologs compared to non-transgenic controls: csp-1, F42G8.7, F41E7.6, Y19D10A.16, C01B4.6, dnj-28, hsp-4, ckb-2, mct-2, lipl-3, and eol-1. Surprisingly, each one of these genes is required for xbp-1s-mediated suppression of tauopathy, suggesting that XBP-1s activates a broad and non-redundant network of cellular mechanisms to reduce tau pathology. Of these, we examined the critical UPRER regulator BiP/hsp-4, the ER resident HSP70 homolog. Hsp-4 loss of function, but not loss of the cognate ER resident DNAJ protein dnj-28, eliminates xbp-1s-mediated suppression of tauopathy. While hsp-4 loss of function exacerbates tau-induced behavioral deficits, tau protein level and phosphorylation are unaffected. High level overexpression of hsp-4 exacerbates tau-induced behavioral deficits and protein accumulation, while moderated hsp-4 overexpression ameliorates this phenotype. Furthermore, caspases also appear to play an important role downstream of XBP-1s: both the XBP-1s target csp-1 and its non-XBP-1s target gene family member ced-3 are required for xbp-1s-mediated suppression of tauopathy. In summary, this data set provides new therapeutic ideas that may be leveraged into novel treatments for tauopathy disorders further development against tauopathy.

